# An erythematous plaque on the forehead

**DOI:** 10.1016/j.jdcr.2025.01.024

**Published:** 2025-02-20

**Authors:** Connor A. Sheehan, Christopher D. Markeson, Sylvia Hsu

**Affiliations:** Department of Dermatology, Lewis Katz School of Medicine at Temple University, Philadelphia, Pennsylvania

**Keywords:** alopecia mucinosa, follicular mucinosis

## Case report

**Fig 1:**
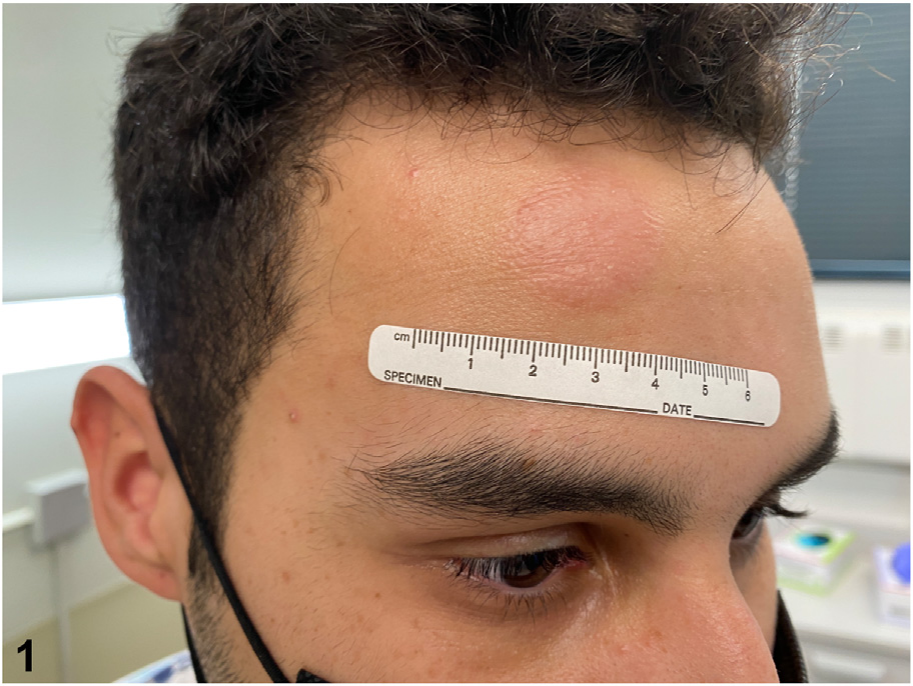

**Fig 2:**
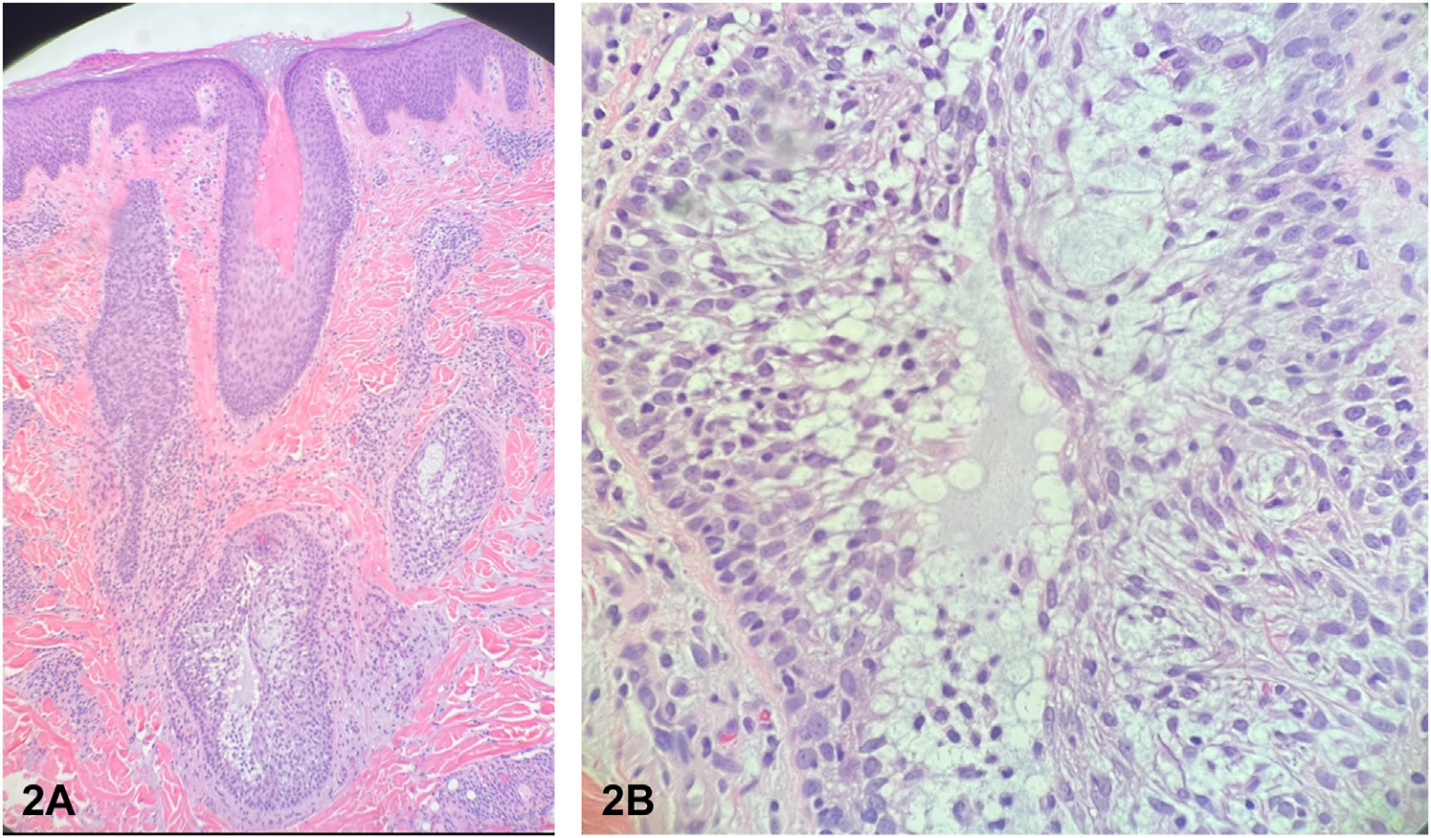

A 20-year-old Hispanic man with no past medical history was concerned about pruritus and erythema of his right forehead. The patient awoke with a pruritic papule, which quickly enlarged to become a plaque. Physical examination revealed a 2.5 cm nonscaly, indurated, erythematous plaque on his right forehead (Fig 1). A 3-mm punch biopsy was performed for histopathological examination (Fig 2, *A* and *B*).

**Question 1: What is your diagnosis?**

A. Alopecia mucinosa
B. Granuloma faciale
C. Lupus erythematosus tumidus
D. Pseudolymphoma
E. Urticaria

**Answers:**

A. Alopecia mucinosa – Correct. Alopecia mucinosa (AM) is a rare disorder characterized histologically by an accumulation of dermal-like mucin (glycosaminoglycans) in the external root sheath of the pilosebaceous unit, resulting in alopecia as follicular growth is hindered and keratinocytes detach from surrounding structures.^1^
B. Granuloma faciale – Incorrect. Granuloma faciale is classically characterized by a solitary, reddish-brown plaque. On histopathology, a dense polymorphous infiltrate of neutrophils and eosinophils, which is mainly present in the upper half of the dermis, does not invade the epidermis and is separated from the epidermis by a zone of normal collagen (grenz zone). Often, there is vasculitis with deposition of fibrinoid material within and around vessel walls.^2^
C. Lupus erythematosus tumidus – Incorrect. Lupus erythematosus tumidus presents with erythematous papules or plaques with histology showing perivascular lymphocytic infiltrate and mucin (glycosaminoglycans) accumulation.
D. Pseudolymphoma – Incorrect. Pseudolymphoma most commonly presents as an erythematous nodule, and the histopathology would show an abundant atypical lymphocytic infiltrate.
E. Urticaria – Incorrect. Urticaria often appears in response to a triggering event with a blanching, erythematous, and pruritic wheal. Histopathology would show interstitial dermal edema, dilated venules with endothelial swelling, and a paucity of inflammatory cells, although urticaria is not typically biopsied.^3^

**Question 2: What is the most common location for this?**

A. Buttocks
B. Chest
C. Head/neck
D. Lower extremities
E. Lower back

**Answers:**

A. Buttocks – Incorrect. Involvement of the buttocks has not been commonly described in the literature.
B. Chest – Incorrect. Lesions on the trunk have been reported, but are less common than on the head and neck.^4^
C. Head/neck – Correct. AM often presents in children or young adults as a single patch or plaque on the head or neck and may be accompanied by alopecia or pruritus.^4^
D. Lower extremities – Incorrect. The upper extremities are a more common location for AM than the lower extremities.^4^
E. Lower back – Incorrect. Lesions on the trunk have been reported but are less common than on the head and neck.^4^

**Question 3: The initial treatment of choice is:**

A. Corticosteroids
B. Dapsone
C. Hydroxychloroquine
D. Minocycline
E. Systemic psoralen plus ultraviolet A light (PUVA) therapy

**Answers:**

A. Corticosteroids – Correct. Treatment of idiopathic AM is aimed at providing symptomatic relief for patients experiencing pruritus. The initial treatment of choice is mild-to-moderate topical corticosteroids but may be substituted with intralesional corticosteroid injections. Additional treatment options include systemic corticosteroids, systemic PUVA therapy, antimalarials, isotretinoin, dapsone, interferon-alpha 2b, and minocycline.^1^ Patients with suspected lymphoma-associated AM should undergo treatment to address the underlying lymphoma, as superficial and topical treatments have been largely ineffective.^4^ Long-term monitoring should be considered in these patients as the association with underlying malignancy is still being investigated.^5^
B. Dapsone – Incorrect. While AM has been shown to be responsive to dapsone, this is not the first-line treatment.^1^^,^^4^
C. Hydroxychloroquine – Incorrect. While AM has been shown to be responsive to antimalarial, like hydroxychloroquine, this is not a first-line treatment.^1^
D. Minocycline – Incorrect. While AM has been shown to be responsive to antibiotics, like minocycline, this is not a first-line treatment.^1^^,^^4^
E. Systemic PUVA therapy – Incorrect. While AM has been shown to be responsive to light therapy, this is not a first-line treatment.^1^^,^^4^
